# Gαi2 regulates the adult myogenesis of masticatory muscle satellite cells

**DOI:** 10.1111/jcmm.17726

**Published:** 2023-03-28

**Authors:** Lin Kong, Yi Fang, Mingyuan Du, Yunlong Wang, Hong He, Zhijian Liu

**Affiliations:** ^1^ The State Key Laboratory Breeding Base of Basic Science of Stomatology (Hubei‐MOST) & Key Laboratory of Oral Biomedicine Ministry of Education, School & Hospital of Stomatology Wuhan University Wuhan China; ^2^ Kunming Medical University School and Hospital of Stomatology Kunming China; ^3^ Yunnan Key Laboratory of Stomatology Kunming China; ^4^ Department of Orthodontics, School and Hospital of Stomatology Wuhan University Wuhan China

**Keywords:** adult myogenesis, Gαi2, masticatory muscle, muscle satellite cell

## Abstract

Although similar to trunk and limb skeletal muscles, masticatory muscles are believed as unique in both developmental origins and myogenesis. Gαi2 has been demonstrated to promote muscle hypertrophy and muscle satellite cell differentiation in limb muscles. However, the effect of Gαi2 on masticatory muscles is still unexplored. This study aimed to identify the role of Gαi2 in the proliferation and differentiation of masticatory muscle satellite cells, further exploring the metabolic mechanism of masticatory muscles. The proliferation rate, myotube size, fusion index of masticatory muscle satellite cells and Pax7, Myf5, MyoD, Tcf21 and Musculin expressions were significantly decreased by Gαi2 knockdown, while in cells infected with AdV4‐Gαi2, the proliferation rate, myotube size, fusion index and Tbx1 expression were significantly increased. Masticatory muscle satellite cells also displayed phenotype transformation as Gαi2 changed. In addition, Gαi2 altered myosin heavy chain (MyHC) isoforms of myotubes with less MyHC‐2A expression in siGαi2 group and more MyHC‐slow expression in AdV4‐Gαi2 group. In conclusion, Gαi2 could positively affect the adult myogenesis of masticatory muscle satellite cells and maintain the superiority of MyHC‐slow. Masticatory muscle satellite cells may have their unique Gαi2‐regulated myogenic transcriptional networks, although they may share some common characteristics with trunk and limb muscles.

## 1 INTRODUCTION

Masticatory muscles consist of masseter, temporalis, medial and lateral pterygoid muscles, playing fundamental roles in oral function, such as sucking, chewing, swallowing, and speech.^1^ In addition, masticatory muscle mass and function are highly associated with facial form and malocclusion.^2^, ^3^ Patients with open bite show less cross‐sectional areas of the masticatory muscles and reduced efficiency of the chewing pattern than normal‐occlusion patients.^4^ Although masticatory and trunk and limb muscles all belong to skeletal muscles sharing similar structures and functions, increasing evidence indicates that notable differences exist among them. Regarding the developmental origin, masticatory muscles derive from the head paraxial mesoderm, while the trunk and limb muscles develop from somites.^5^ In addition, masticatory muscles present smaller fibres, a higher percentage of hybrid fibres, and more type I, II and foetal myosin heavy chain (MyHC) isoforms compared to the trunk and limb muscles.^1^ Furthermore, regulatory pathways controlling myogenesis in the masticatory muscles are distinct from those in the trunk and limb muscles. Previous studies discovered that Pax3 and Myf5 double mutant mice remained normal masticatory muscles but failed to develop trunk muscles.^6^ In contrast, masticatory muscles were eliminated, whereas other skeletal muscles were unaffected in mice lacking both MyoR and capsulin.^7^

It is widely believed that masticatory muscles have a huge regenerative capacity to keep their homeostasis, maintaining healthy muscle mass and function, due to the fundamental functions of muscle satellite cells.^8^ Muscle satellite cells are quiescent myogenic cells, lying on the top of muscle fibres and generally keeping quiescent in adult skeletal muscles, but be able to activate and proliferate to regenerate, repair and remodel muscle tissues in responding to physiological and pathological changes.^9^

Gαi2 is a subtype of G proteins which are a family of proteins acting as molecular switches inside cells.^10^ Typical G protein‐coupled receptors (GPCRs) such as β2‐adrenergic receptors, can transduce signals through the Gαi‐linked Gβγ complex in many cell types, independently of activating cyclic AMP through Gαs proteins.^11^, ^12^, ^13^ Previous studies revealed that activated Gαi2 promoted skeletal myotube growth through PKC‐GSK3β and mTOR‐p70S6K pathways.^14^ In addition, Gαi2 was also confirmed to induce the activation of limb muscle satellite cells via PKC‐GSK3β pathway and HDAC inhibition.^15^ However, the role of Gαi2 in masticatory muscles is still uncertain. This study aimed to identify the role of Gαi2 in the proliferation and differentiation of masticatory muscle satellite cells, further exploring the metabolic mechanism of masticatory muscles. siRNA‐mediated Gαi2 knockdown and adenoviral‐mediated Gαi2 overexpression were conducted and the changes of cell proliferation rate, myotube size, fusion index, transcription factor expressions, cell phenotypes and MyHC isoforms were detected in this study.

## 2 MATERIALS AND METHODS

### 2.1 The isolation, culture and identification of primary masticatory muscle satellite cells

A total of 30 adult male C57BL10 mice (8–12 weeks old) were purchased from Hubei Research Center of Laboratory Animals and fed at a specific pathogen free (SPF)‐class housing of laboratory in Hubei Research Center of Laboratory Animals (Wuhan, Hubei Province, China). The mice were all healthy and had no trauma, being provided with free access to normal chow and water. Animal studies were approved by the Ethics Committee of Wuhan University School and Hospital of stomatology. All procedures were proceeded according to the institutional guidelines on the use of animals in research.

Primary masticatory muscle satellite cells were isolated from masseters of C57BL10 mice as previously reported.^16^, ^17^, ^18^ In brief, mice were sacrificed by cervical dislocation, and masseters were dissected and digested with collagenase I (Sigma‐Aldrich) in Dulbecco's modified Eagle medium (DMEM) containing sodium pyruvate for 1 h. Myofibers and associated muscle satellite cells were cultured in growth medium 1 (GM1) (DMEM containing sodium pyruvate supplemented with 10% horse serum (Hyclone), 0.5% chicken embryonic extract, 4 mM L‐glutamine and 1% penicillin/streptomycin) for 72 h. Then, myofibers were removed and muscle satellite cells were cultured in growth medium 2 (GM2) (DMEM containing sodium pyruvate supplemented with 20% FBS (Hyclone), 10% horse serum, 0.5% chick embryo extract, and 1% penicillin/streptomycin) for 48 h. For activation and proliferation analysis, masticatory muscle satellite cells were cultured in GM2 for another 48 h. For studies of differentiation efficiency, GM2 was replaced with differentiation medium (DM) (DMEM containing sodium pyruvate supplemented with 2% horse serum, 0.5% chick embryo extract and 1% penicillin/streptomycin), and cells were cultured for additional 48 h. Cultures were maintained in a humidified 5% CO_2_ incubator at 37°C throughout all of the experimental procedures.

### 2.2 siRNA‐mediated Gαi2 knockdown and adenoviral‐mediated Gαi2 overexpression

siGαi2 and siRNA negative control (siCtrl) construct were purchased from Genepharma. Masticatory muscle satellite cells cultured in GM2 were transfected with siGαi2 and siCtrl construct using Lipofectamine® 2000 Reagent (Invitrogin).

Adenoviral constructs AdV4‐Gαi2 and control empty vector AdV4‐NC were generated, amplified and purified by Genepharma. Masticatory muscle satellite cells cultured in GM2 were infected for 24 h with AdV4‐Gαi2 or AdV4‐NC (multiplicity of infection = 200). After infection, the cells were cultured in GM2 or DM for another 48 h.

### 2.3 Proliferation assay

The cell proliferation after Gαi2 knockdown or overexpression was evaluated by Cell‐Light™ EdU Apollo®488 In Vitro Imaging Kit (RiboBio) according to the manufacturer's instructions. Briefly, cells were labelled with 5‐ethynyl‐2′‐deoxyuridine (Edu, RiboBio) at a final concentration of 50 μM for 2 h. Then, the absorbance was determined at a wavelength of 488 nm.

### 2.4 Myotube size analysis and fusion assay

To determine changes in myotube size and fusion index, myotubes were washed with cytoskeleton stabilising buffer (CSB) containing 80 mM PIPES, 5 mM EGTA, 1 mM MgCl_2_, PEG 35000 (40 g/L; polyethylene 140 glycol, molecular weight, 35,000) in distilled water with a pH of 7.4. Cells were then fixed with 4% paraformaldehyde (PFA) in CSB for 15 min and permeabilized with 0.2% Triton in CSB at room temperature. After the nonspecific binding was blocked with 10% goat serum (Gibco), myotubes were incubated with monoclonal antibody MF20 which recognized all MyHC isoforms (1:100, DSHB) at 4°C overnight. Subsequently, cells were stained with Alexa488 and 4,6‐diami‐dino‐2‐phenylindole (DAPI) (Aspen), and mounted in Vectashield mounting medium (biosharp).

For the measurement of myotube diameter, three pictures were taken in each well. Thirty largest myotubes from each picture were selected and measured by Image‐Pro Plus 6.0. After dividing myotubes into thirds, the myotube diameter was calculated as the mean of distances between the midpoints of each portion. To analyse fusion index, the nuclei of 60–90 myotubes in each well were counted to obtain the average number of nuclei per myotube.

### 2.5 Immunostaining

For immunofluorescent detection of different phenotypes of muscle satellite cells under different conditions, the following antibodies were used: rabbit polyclonal anti‐MyoD (1:100, Santa Cruz Biotechnology) and mouse monoclonal anti‐Pax7 (1:100, DSHB). After fixation with 4% PFA, cells were incubated with primary antibodies at 4°C overnight, nonspecific binding was blocked with 10% goat serum (Gibco) and the primary antibodies were visualized using appropriate species‐specific 488 and 549 fluoro‐chrome‐conjugated secondary antibodies (1:100, Abbkine), stained with DAPI (Aspen), and mounted in Vectashield mounting medium (biosharp).

### 2.6 RNA isolation and analysis

RNA was isolated with Total RNA kit I (OMEGA) and cDNA was prepared from 200 ng RNA according to the manufacturer's instructions (Thermo Fisher Scientific). The relative level of gene expression was determined by quantitative real‐time polymerase chain reaction (qRT‐PCR) using a 7900 HT Fast Real‐Time PCR System (Applied Biosystems). Primers used for detection are listed in Table 1.^19^, ^20^, ^21^, ^22^ All the MyHC types existing in mouse masseter were detected to explore the change of muscle fibre types (Table 2). Glyceraldehyde‐3‐phosphate dehydrogenase (GAPDH) was used as the internal control. Relative transcript abundance was normalized to the amount of genes in control group and quantitated by the 2^−ΔΔCT^ method.

**TABLE 1:** Primers for qRT‐PCR.

Genes	Forward primer	Reverse primer
Pax7	5′ CCGTGTTTCTCATGGTTGTG 3′	5′ GAGCACTCGGCTAATCGAAC 3′
Myf5	5′ GGCTGTAATAGTTCTCCACCTGTT 3′	5′ TGTATCCCCTCACCAGAGGAT 3′
MyoD	5′ TGCAGTCGATCTCTCAAAGCACC 3′	5′ GCAGGCTCTGCTGCGCGACC 3′
Tcf21	5′ AGACCTTTAAGGGGCTGGAG 3′	5′ GATTTTCGCCGTTTCTTGG 3′
Musculin	5′ ACATTCACCCAGTCAACCTG 3′	5′ CCACTTCCTTCAGGTCATTCTC 3′
Tbx1	5′ GCTGTGGGACGAGTTCAATC 3′	5′ ACGTGGGGAACATTCGTCT 3′
Myh1	5′ GAGGGACAGTTCATCGATAGCAA 3′	5′ GGGCCAACTTGTCATCTCTCAT 3′
Myh2	5′ AGGCGGCTGAGGAGCACGTA 3′	5′ GCGGCACAAGCAGCGTTGG 3′
Myh3	5′ CTTCACCTCTAGCCGGATGGT 3′	5′ AATTGTCAGGAGCCACGAAAAT 3′
Myh4	5′ CACCTGGACGATGCTCTCAGA 3′	5′ GCTCTTGCTCGGCCACTCT 3′
Myh6	5′ CCAACACCAACCTGTCCAAGT 3′	5′ AGAGGTTATTCCTCGTCGTGCAT 3′
Myh7	5′ CTCAAGCTGCTCAGCAATCTATTT 3′	5′ GGAGCGCAAGTTTGTCATAAGT 3′
Myh8	5′ CAGGAGCAGGAATGATGCTCTGAG 3′	5′ AGTTCCTCAAACTTTCAGCAGCCAA 3′
GNAI2	5′ TTGGCCGCTCACGAGAATA 3′	5′ GCTGACCACCCACATCAAACA 3′
GAPDH	5′ CCACTCTTCCACCTTCG 3′	5′ GTGGTCCAGGGTTTCTTAC 3′

**TABLE 2:** Composition of MyHC isoforms in mouse masseter.

Genes	MyHC isoforms
Myh1 (2X‐MyHC)	Typical fast isoforms
Myh2 (2A‐MyHC)	
Myh4 (2B‐MyHC)	
Myh7 (slow‐MyHC)	Slow isoform
Myh3 (emb‐MyHC)	Developmental isoforms
Myh8 (neo‐MyHC)	
Myh6 (cardiac MyHC)	Other isoform

### 2.7 Statistical analysis

All the experiments in this study were independently performed three times. Data was expressed as mean ± standard deviation. Differences between two groups were evaluated using paired Student's *t*‐test. *p*‐value < 0.05 was considered as statistically significant. Statistical analysis was conducted with SPSS 13.0 statistical software (IBM Corp).

## 3 RESULTS

**FIGURE 1:**
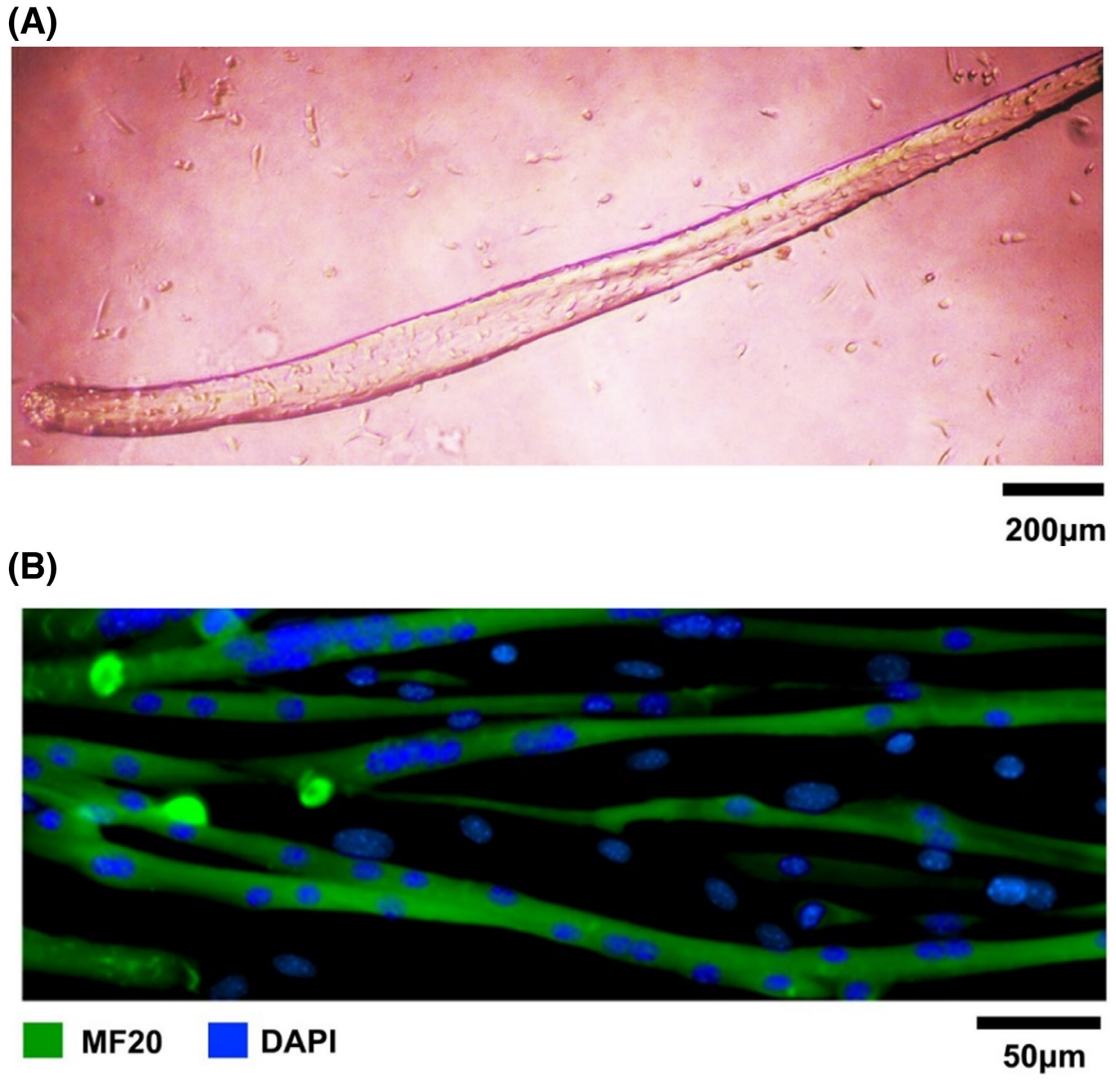
Primary masticatory muscle satellite cells. (A) Masticatory muscle satellite cells were found to scatter around myofibers after culturing in GM1 for 72 h. (B) Immunofluorescence confirmed that masticatory muscle satellite cells differentiated into long and multinuclear myotubes.

Primary masticatory muscle satellite cells were successfully isolated, cultured and differentiated into long and multinuclear myotubes (Figure 1).

### 3.1 Knockdown of Gαi2 inhibits the activation, proliferation and differentiation of masticatory muscle satellite cells

**FIGURE 2:**
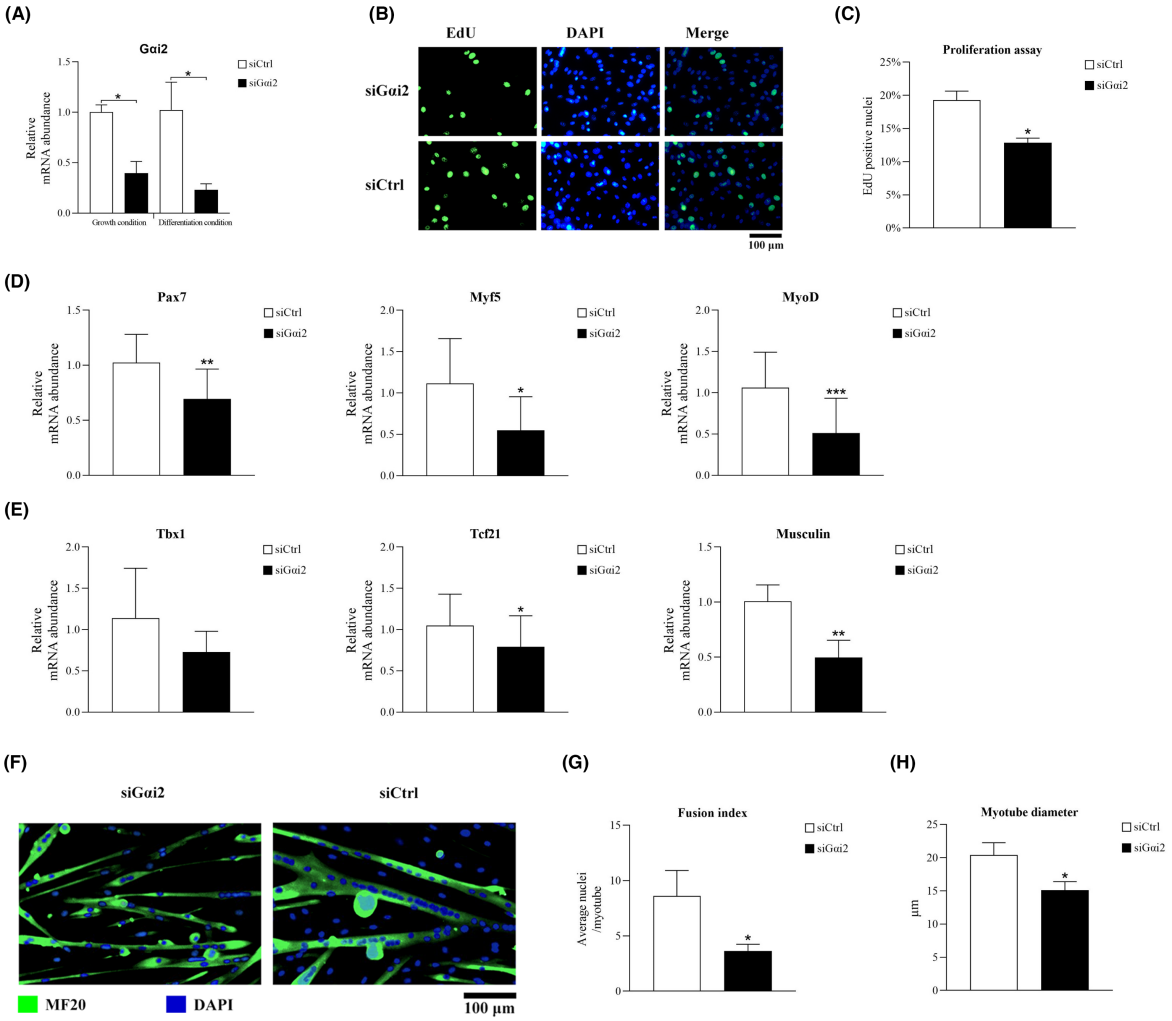
Knockdown of Gαi2 inhibits the activation, proliferation and differentiation of masticatory muscle satellite cells. (A) Knockdown of Gαi2 in the siGαi2 group was confirmed by qRT‐PCR. (B, C) Knockdown of Gαi2 inhibited the proliferation rate of masticatory muscle satellite cells. (D, E) Under growth condition, Pax7, Myf5, MyoD, Tcf21 and Musculin expressions were significantly decreased while Tbx1 expression was not significantly affected in the siGαi2 group. (F–H) Knockdown of Gαi2 reduced the myotube size and the fusion index of masticatory muscle satellite cells. *n* = 3 independent experiments. *p < 0.05; **p < 0.01; ***p < 0.001.

Fluorescence‐labelled siRNA was used to suppress the expression of Gαi2 in masticatory muscle satellite cells (Figure S1). Knockdown of Gαi2 in the siGαi2 group was confirmed by qRT‐PCR under both growth and differentiation conditions (Figure 2A). Under growth condition, cells transfected with siGαi2 showed a significantly lower proliferation rate than the control group (Figure 2B,C). The mRNA levels of quiescence marker (Pax7), and activation marker (Myf5), together with the differentiation marker (MyoD) were significantly decreased in the siGαi2 group (Figure 2D). Some transcription factors regulating embryonic myogenesis of masseter, such as Tcf21 and Musculin, were also negatively affected by Gαi2 knockdown (Figure 2E). Under differentiation condition, the myotube size and the fusion index were significantly lower in siGαi2 group compared with the control group (Figure 2F–H).

### 3.2 Overexpression of Gαi2 promotes the activation, proliferation and differentiation of masticatory muscle satellite cells

**FIGURE 3:**
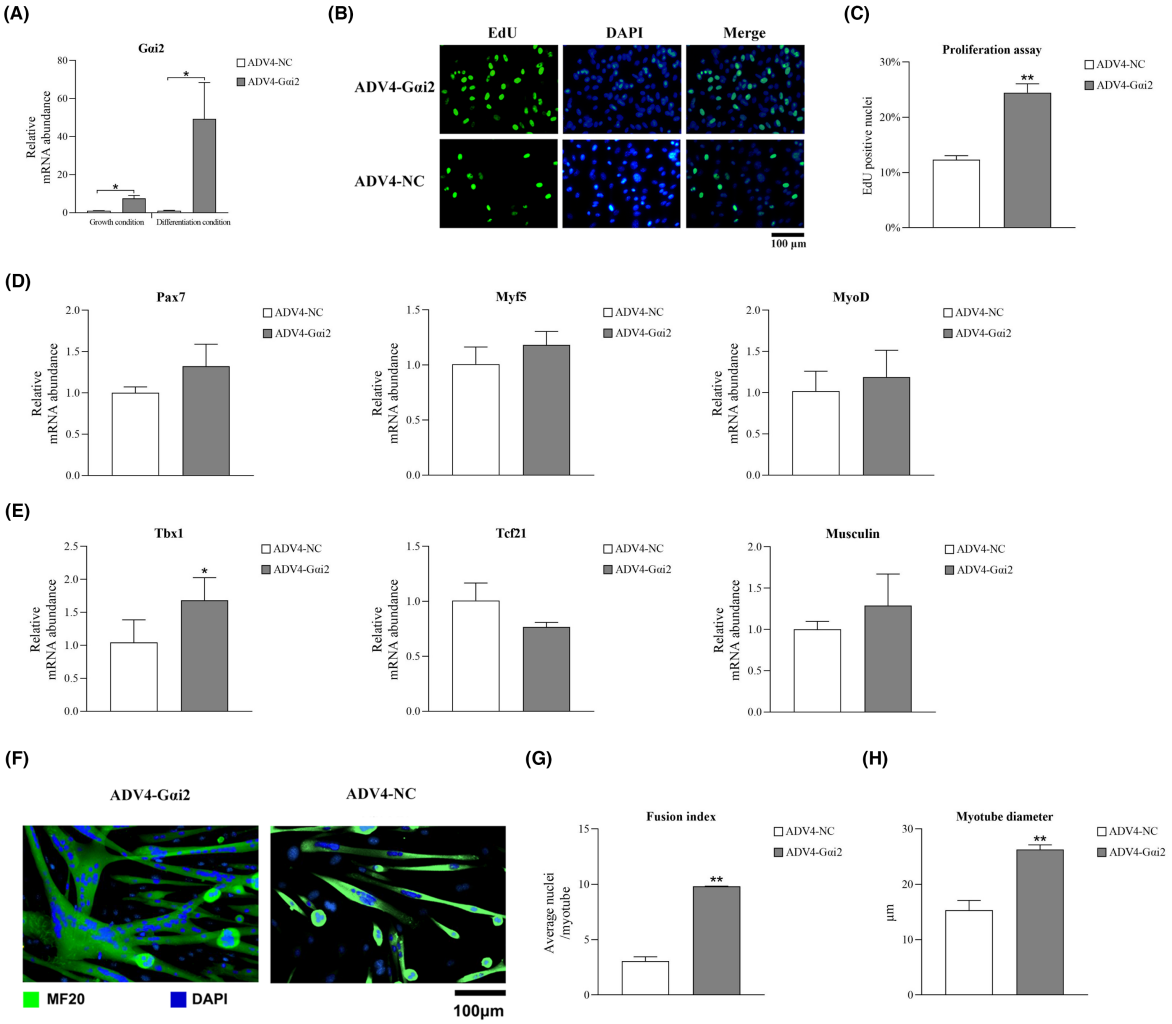
Overexpression of Gαi2 promotes the activation, proliferation and differentiation of masticatory muscle satellite cells. (A) Overexpression of Gαi2 in the AdV4‐Gαi2 group was confirmed by qRT‐PCR. (B, C) Overexpression of Gαi2 promoted the proliferation rate of masticatory muscle satellite cells. (D, E) Under growth condition, Tbx1 expression was significantly increased while Pax7, Myf5, MyoD, Tcf21 and Musculin expressions had no significant changes in the AdV4‐Gαi2 group. (F–H) Overexpression of Gαi2 increased the myotube size and the fusion index of masticatory muscle satellite cells. *n* = 3 independent experiments. *p < 0.05; **p < 0.01.

Adenovirus was employed to overexpress Gαi2 in masticatory muscle satellite cells under growth and differentiation conditions (Figure 3A). Under growth condition, a significantly higher ratio of Edu‐positive cells was noted in the AdV4‐Gαi2 group than that in the AdV4‐NC group (Figure 3B,C). Overexpressing Gαi2 significantly increased Tbx1 expression, while the mRNA expression level of Pax7, Myf5, MyoD, Tcf21 and Musculin had no significant changes (Figure 3D,E). Under differentiation condition, muscle satellite cells differentiated and fused into significantly larger myotubes with more nuclei in the AdV4‐Gαi2 group compared to the control group (Figure 3F–H).

### 3.3 Gαi2 induces phenotype transformation of masticatory muscle satellite cells

**FIGURE 4:**
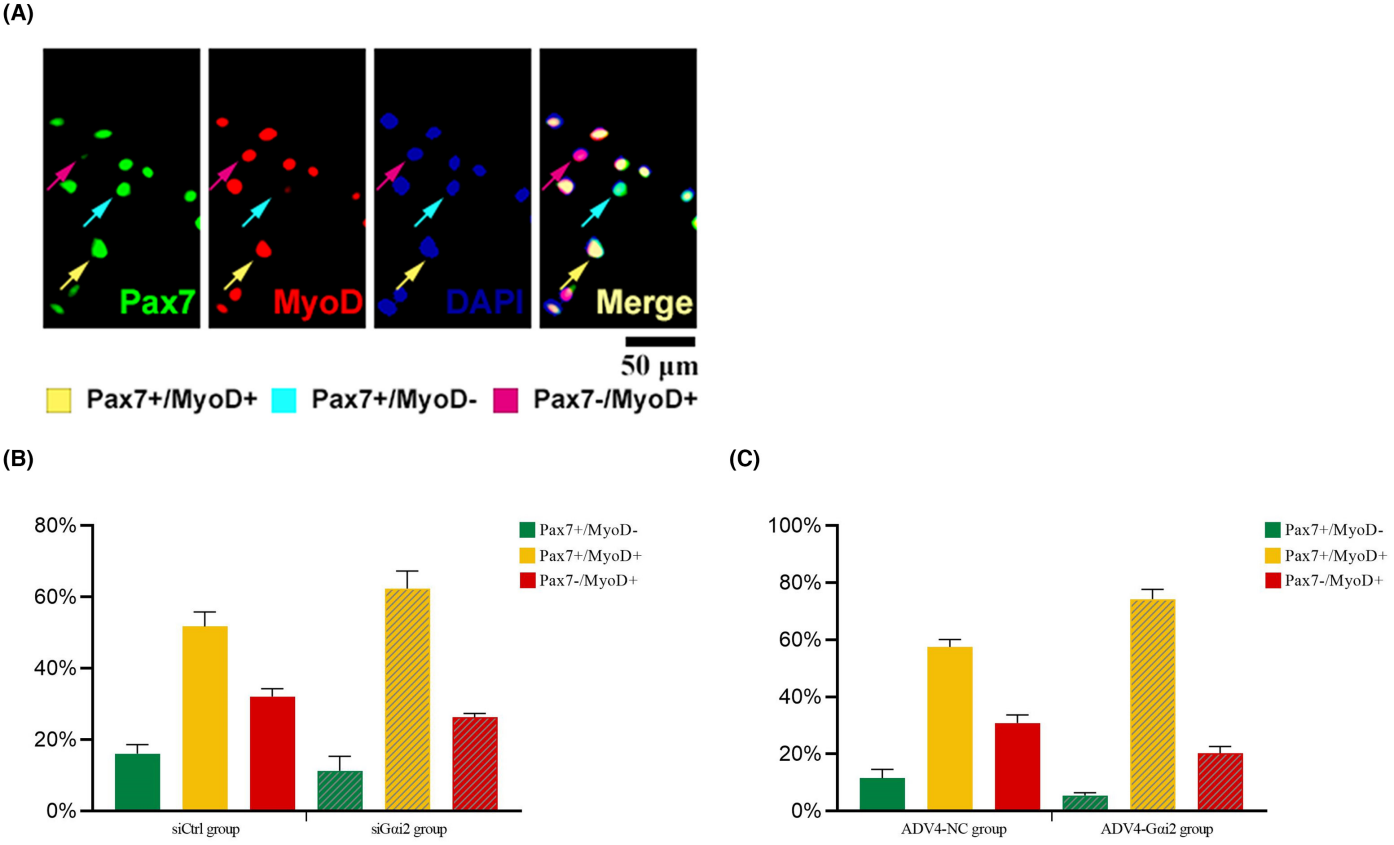
Gαi2 changes the percentage of different phenotypes of masticatory muscle satellite cells. (A) Three phenotypes of masticatory muscle satellite after being immunostained of Pax7/MyoD. (B) siGαi2 group showed a higher percentage of Pax7+/MyoD+ cells and a lower percentage of Pax7+/MyoD− and Pax7−/MyoD+ cells than the siCtrl group. (C) AdV4‐Gαi2 group had a higher percentage of Pax7+/MyoD+ cells and a lower percentage of Pax7+/MyoD− and Pax7−/MyoD+ cells compared to the AdV4‐NC group. Three pictures were taken in each well and the average percentage of different cell phenotypes was calculated. *n* = 3 independent experiments.

Masticatory muscle satellite cells were immunostained for Pax7/MyoD after knocking down or overexpressing Gαi2 to explore the role of Gαi2 in the functional status of cells (Figure 4A). Muscle satellite cells coexpressing both Pax7 and MyoD (Pax7+/MyoD+) were regarded as activated state, that is, a state before making the decision to undergo self‐renewing or differentiating process. Muscle satellite cells expressing only Pax7 (Pax7+/MyoD−) were going to be self‐renewed, and those expressing only MyoD (Pax7−/MyoD+) were expected to undergo differentiation.^16^, ^23^

In experiments of knocking down of Gαi2, the percentage of different phenotypes of masticatory muscle satellite cells was calculated and the percentage of Pax7+/MyoD+ cells was found to be higher in the siGαi2 group (Pax7+/MyoD+ 62.37 ± 4.00%) compared with the siCtrl group (Pax7+/MyoD+ 51.78 ± 3.31%). By contrast, the percentage of both Pax7+/MyoD− and Pax7−/MyoD+ cells were obviously lower in the siGαi2 group (Pax7+/MyoD− 11.30 ± 3.33%, Pax7−/MyoD+ 26.33 ± 0.86%) than the siCtrl group (Pax7+/MyoD− 16.09 ± 2.09% Pax7−/MyoD+ 32.13 ± 1.80%) (Figure 4B).

In experiments of over‐expression of Gαi2, there were less cells with Pax7+/MyoD− and Pax7−/MyoD+ in AdV4‐Gαi2 group (Pax7+/MyoD− 5.42 ± 0.86%, Pax7−/MyoD+ 20.28 ± 1.94%) compared with AdV4‐NC group (Pax7+/MyoD− 11.58 ± 2.52%, Pax7−/MyoD+ 30.90 ± 2.30%). Additionally, a greater number of Pax7+/MyoD+ cells were found in the AdV4‐Gαi2 group (Pax7+/MyoD+ 74.30 ± 2.80%) compared with the AdV4‐NC group (Pax7+/MyoD+ 57.52 ± 2.11%) (Figure 4C).

### 3.4 Gαi2 alters MyHC transition in masticatory muscle myotubes

The most accepted methods to classify muscle fibre types are based on specific myosin profiles, especially the MyHC isoform complement.^24^ The mRNA expressions of different MyHC were tested when Gαi2 was suppressed or over‐expressed to explore the possible role of Gαi2 in determining masticatory muscle fibre types.

**FIGURE 5:**
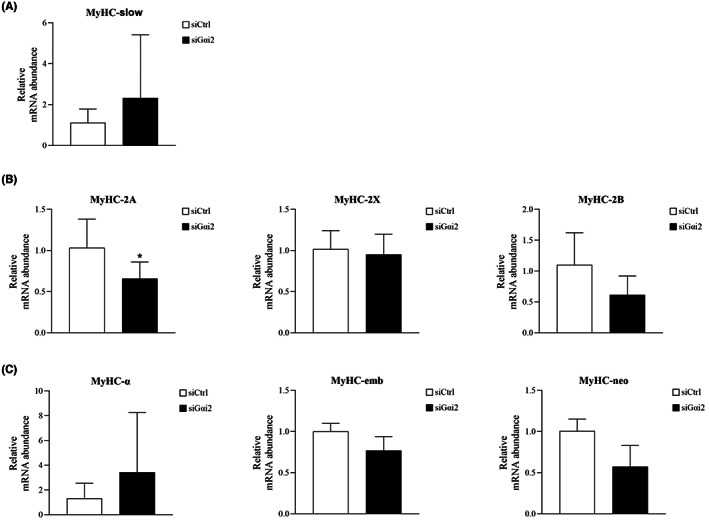
The changes of MyHC expression after Gαi2 down‐regulation. (A) MyHC‐slow was not significantly influenced in the siGαi2 group. (B) MyHC‐2A was significantly decreased and MyHC‐2X and MyHC‐2B had no significant changes in the siGαi2 group. (C) MyHC‐α, MyHC‐emb and MyHC‐neo were not significantly changed in the siGαi2 group. *n* = 3 independent experiments. *p < 0.05.

**FIGURE 6:**
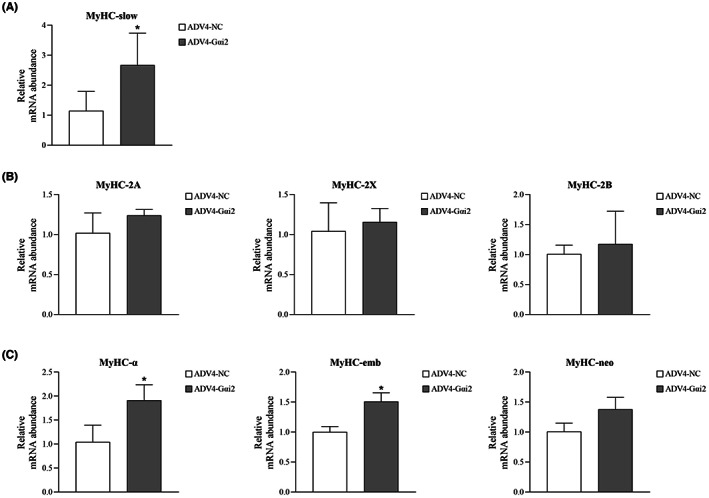
The changes of MyHC expression after Gαi2 upregulation. (A) MyHC‐slow was significantly increased in the AdV4‐Gαi2 group. (B) MyHC‐2A, MyHC‐2X and MyHC‐2B had no significant changes in the AdV4‐Gαi2 group. (C) MyHC‐α and MyHC‐emb were upregulated and MyHC‐neo was not significantly influenced in the AdV4‐Gαi2 group. *n* = 3 independent experiments. *p < 0.05.

Under differentiation condition, when Gαi2 was suppressed in masticatory muscle satellite cells, the mRNA expression of MyHC‐2A was significantly decreased but other types of MyHC had no significant changes (Figure 5A–C). In contrast, Gαi2 overexpression significantly upregulated gene expressions of MyHC‐slow, MyHC‐α and MyHC‐emb, but had no significant effect on MyHC‐2 expression (Figure 6A–C).

## 4 DISCUSSION

Masticatory muscle mass and function, similar to other skeletal muscles, are maintained by signalling networks that control the proliferation and differentiation of cells as well as the synthesis and degradation of proteins. There is a strong correlation between masticatory muscles and dentofacial deformities. Masticatory muscle hyperfunction could increase sutural growth and affect the growth rotation pattern of the mandible, influencing the transversal and vertical dimensions of craniofacial bones.^25^ In addition, muscle fibre types are also reported to affect jawbone morphology, with higher proportion of type II fibres in short‐face patients and lower proportion of type II fibres in long‐face patients.^26^ Muscles and bones are in close crosstalk, it is important to maintain their homeostasis.^27^ Current evidence suggests that muscle satellite cells are playing important roles in the homeostasis of muscle tissues. Muscle satellite cells normally keep as quiescent in adult muscles but are able to be activated in response to chemical and physical changes. Once confronting those stimuli, muscle satellite cells are activated, partially self‐renewing to keep their quantities, and partially differentiating into myoblasts to form muscle tissues.^23^ This postnatal myogenesis process is governed by a gene regulatory network that includes the paired‐domain transcription factors (Pax3 and Pax7) and myogenic regulatory factors (MyoD, Myf5, myogenin and Mrf4).^28^

Gαi2, as a required component of various GPCR actions, plays an important part in regulating many biological processes including development and differentiation. For example, β2‐adrenoceptor agonists have been indicated in regulating skeletal muscle mass.^29^ In addition, GPR56, a transcriptional target of peroxisome proliferator‐activated receptor gamma coactivator 1‐alpha 4 (PGC‐1α4), is also involved in muscle hypertrophy.^30^ Moreover, β2‐adrenergic receptors and GPR56 are both reported to initiate downstream signals through Gαi.^13^, ^31^ Gαi2 mutant mice develop ulcerative colitis and present growth retardation and decreased thymocyte maturation and function.^32^ Previous studies also demonstrated that Gαi2‐mediated signalling could promote muscle hypertrophy, myoblast differentiation and muscle regeneration in limb skeletal muscles through PKC‐GSK3β and mTOR‐p70S6K pathways.^14^, ^15^ However, its role in masticatory muscles is still unexplored. Since masticatory muscles are embryonic origined differently and biochemical behaved differently from trunk and limb skeletal muscles,^27^ it is essential to verify whether or not Gαi2 has similar effects on masticatory muscle satellite cells, and explore the specific regulatory pathways to further our understanding in the metabolic mechanism of masticatory muscles. As far as our best knowledge, this is the first report focusing on the role of Gαi2 signalling in masticatory muscle homeostasis.

In this study, we explored the possible role of Gαi2 in the proliferation and differentiation of masticatory muscle satellite cells by knocking down and overexpressing Gαi2. Our results showed that Gαi2 can positively affect the proliferation rate, myotube diameter and fusion index of masticatory muscle satellite cells. The mRNA expression level of some transcription factors, cell phenotypes and muscle fibre types also displayed alteration as Gαi2 changed. Since proteins we extracted from primary culture of masticatory muscle satellite cells were insufficient to support a reliable Western blot analysis and significant heterogeneity may arise when culturing primary cells from different mice together, we did not obtain a reliable result of Western blot analysis and the protein level of transcription factors and MyHC isoforms was not displayed in this study.

First, we focused on three crucial transcription factors associated with adult myogenesis of skeletal muscle satellite cells, including Pax7, MyoD and Myf5. Pax7 regulates the expansion and differentiation of muscle satellite cells during both neonatal and adult myogenesis.^8^ MyoD and Myf5 are believed to determine the concatenate differentiation processes from skeletal muscle satellite cells to muscle fibres.^33^, ^34^ Moreover, Pax7 is confirmed to directly regulate the expression of Myf5.^35^ In this study, results of qRT‐PCR suggested that the suppressed Gαi2 significantly downregulated the expression of Pax7, Myf5 and MyoD which was consistent with the findings in limb muscle satellite cells.^15^ However, when Gαi2 was overexpressed, the expression of Pax7, Myf5 and MyoD were not significantly influenced. This phenomenon was different from a previous study of limb muscles.^14^ These results suggested that Gαi2 was essential to the myogenic process of masticatory muscle satellite cells, but the upregulating effect from Gαi2 was not as strong as those in trunk and limb muscle satellite cells. In addition, we also examined the expression of three head muscle myogenesis‐specific transcription factors, including Tbx1, Tcf21 and Musculin.^36^, ^37^ Tbx1 is required for the normal expression of Myf5 and MyoD.^38^ Tcf21 and Musculin are demonstrated to be the direct upstream activators of both Myf5 and MyoD in branchiomeric myogenesis.^39^ Results of qRT‐PCR showed that Tcf21 and Musculin expressions were significantly decreased in the siGαi2 group, in line with the downregulation of Myf5 and MyoD mentioned above. Meanwhile, Tbx1 expression showed a significant increase in cells infected with AdV4‐Gαi2, indicating that these three transcription factors may also be involved in the myogenic transcriptional networks of Gαi2.

Skeletal muscle satellite cells may at different functional status undergoing divergent cellular fates: Pax7+/MyoD+ cells in an activated state, Pax7+/MyoD− cells retaining self‐renewal and Pax7−/MyoD+ cells with myogenic differentiation capacity.^40^ In this study, the majority of masticatory muscle satellite cells were Pax7+/MyoD+ phenotype, and Pax7+/MyoD− cells were the least in both experiment and control groups. When Gαi2 was knocked down, the proliferation capability of masticatory muscle satellite cells seemed to be most severely inhibited. Whereas, the number of activated cells increased the most in the group over‐expressing Gαi2, indicating that masticatory muscle satellite cells were actively proliferating and going to differentiate very soon.

From the perspective of physiology, skeletal muscle fibres could change their structural and functional properties in reaction to different circumstances. The expression of MyHC isoforms is regarded as the most acceptable criterion to classify skeletal muscle fibre types and the transition between different types of MyHC represents the remodelling of muscles.^24^, ^41^ When Gαi2 was suppressed in masticatory muscle myotubes, MyHC‐2A was significantly downregulated, but the MyHC‐slow was not affected. While in the masticatory muscle myotubes overexpressing Gαi2, the abundance of MyHC‐slow rather than MyHC‐2 was significantly increased. Therefore, the shift direction of myosin expression seemed to always follow the obligatory pathway: MyHC‐2B → MyHC‐2X → MyHC‐2A → MyHC‐slow. Functions of Gαi2 in regulating myosin expression of masticatory muscle myotubes can be reasonably speculated as maintaining the superiority of MyHC‐slow or slow fibre type. Interestingly, our results were inconsistent with two previous studies of limb muscles in which Gαi2 preferred to predominantly maintain MyHC‐2.^14^, ^15^ This deviation may be attributed to the difference in embryonic backgrounds of muscle‐derived stems. In this study, masseter muscles deriving from cranial paraxial mesoderm were used to extract primary muscle satellite cells, while those two studies mentioned above used C2C12 cell line from thigh muscles and primary muscle satellite cells from hind limb muscles, which both belonged to limb muscles deriving from somites.^14^, ^15^ Another possible explanation is the different relationship between muscle fibre type and fibre size among different skeletal muscles. Slow‐type fibres have a larger fibre cross‐sectional area than the fast‐type fibres in masseter muscles while the condition is reversed in the trunk and limb muscles.^42^ Furthermore, MyHC‐α, also known as MyH6, predominantly expressed in cardiac muscles,^43^ was found to be significantly upregulated by Gαi2 overexpression in masticatory muscles. These findings provided insights into a hypothesis that Gαi2 may participate in the regulation of lineage commitment among trunk and limb muscle satellite cells, masticatory muscle satellite cells and cardiac muscle satellite cells. Given the knowledge that trunk and limb muscles are primarily affected by critical illness myopathy (CIM), which is characterized by muscle wasting and myogenesis defect, while masticatory muscles and cardiac muscles are spared or less affected, Gαi2 may play roles in increasing masticatory muscles' resistance against CIM.^44^ Further studies are needed to confirm this speculation and elucidate the underlying mechanisms.

In summary, our study demonstrated that Gαi2 could positively affect the adult myogenesis of masticatory muscle satellite cells and maintain the superiority of MyHC‐slow. Masticatory muscle satellite cells may have their unique Gαi2‐regulated myogenic transcriptional networks, although they may share some common characteristics with trunk and limb muscles.

## AUTHOR CONTRIBUTIONS

**Lin Kong:** Conceptualization (supporting); data curation (equal); formal analysis (equal); investigation (equal); writing – original draft (equal). **Yi Fang:** Conceptualization (supporting); data curation (equal); formal analysis (equal); investigation (equal); writing – original draft (equal). **Mingyuan Du:** Funding acquisition (supporting); methodology (equal); resources (equal); writing – review and editing (supporting). **Yunlong Wang:** Methodology (equal); resources (equal); writing – review and editing (supporting). **Hong He:** Conceptualization (supporting); supervision (equal); writing – review and editing (supporting). **Zhijian Liu:** Conceptualization (lead); funding acquisition (lead); supervision (equal); writing – review and editing (lead).

## CONFLICT OF INTEREST STATEMENT

The authors declare no conflict of interest.

## Supporting information


Figure S1.

## Data Availability

The data that support the findings of this study are available from the corresponding author upon reasonable request.
