# Supplementary material for: Gαi2 regulates the adult myogenesis of masticatory muscle satellite cells
Source: J Cell Mol Med. 2023 Mar 28;27(9):1239–49. doi: 10.1111/jcmm.17726 (PMC10148056; doi:10.1111/jcmm.17726)
Supplement: Supplementary file 1 — Figure S1. [file JCMM-27-1239-s001.docx]

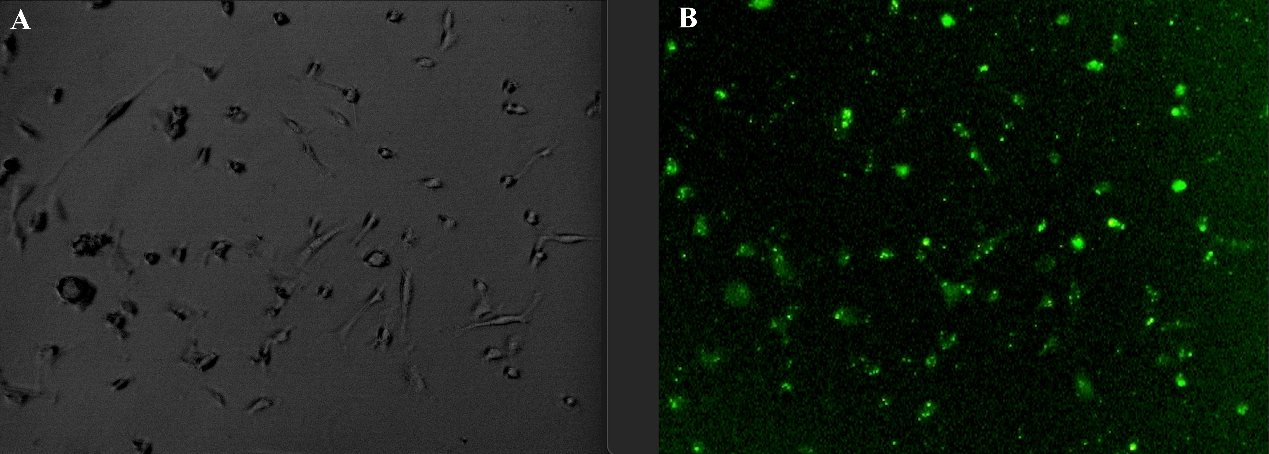


**Supplementary Figure 1.** Fluorescence-labeled siRNA is used to suppress the expression of Gαi2. After transfecting with fluorescence-labeled siRNA for 24 hours, the morphology of masticatory muscle satellite cells (A) and the distribution of fluorescence indicator (B) under the same field of view confirmed the successful transfection of siRNA.
